# Perceived correctional climate and suicidal ideation among inmates in Nigerian correctional centres

**DOI:** 10.3389/fpsyg.2026.1938554

**Published:** 2026-09-28

**Authors:** Happiness Okoka, Emeka E. Obioha

**Affiliations:** Department of Social Sciences, Faculty of Law Humanities and Social Sciences, Walter Sisulu University, Mthatha, South Africa

**Keywords:** correctional centres, correctional climate, inmate mental health, Nigeria, suicidal ideation

## Abstract

This study examined the relationship between perceived correctional climate factors (activity, emotional feedback, freedom, privacy, safety, social structure, and support) and suicidal ideation among inmates in Nigerian correctional Centres. Using a descriptive, cross-sectional survey design, data were collected from a purposive sample of 210 inmates across two major correctional facilities in southern Nigeria. The prison environment inventory (PEI) was used to assess seven dimensions of correctional climate, while the Beck scale for suicide ideation (BSS) measured suicidal ideation. Data were analyzed using Pearson product–moment correlation and multiple linear regression. The results showed that correctional climate factors were jointly significantly associated with suicidal ideation, accounting for 48.3% of its variance, *R*^2^ = 0.483, *F* (7, 202) = 26.94, *p* < 0.001. Individually, more favourable perceptions of privacy (*β* = −0.34, *p* < 0.001), safety (*β* = −0.29, *p* < 0.001), and emotional feedback (*β* = −0.22, *p* = 0.004) were significantly associated with lower suicidal ideation. Institutional support (*β* = −0.19, *p* = 0.015) and structured activities (*β* = −0.14, *p* = 0.049) were also significantly inversely related to suicidal ideation, whereas perceived freedom and social structure were not statistically significant. The findings suggest that perceived correctional climate is significantly related to suicidal ideation among inmates, with privacy and safety emerging as particularly important environmental correlates. The study recommends integrating mental health screening with environmental and institutional measures that strengthen privacy, safety, support, and meaningful activities within correctional settings.

## Introduction

Suicidal ideation is an important mental health concern within correctional settings, where individuals may experience psychological distress associated with loss of liberty, social disruption, restricted autonomy, and adaptation to institutional life (Amoke et al., 2020; Fentahun et al., 2024). Within such settings, correctional climate refers to the perceived psychological, social, and physical characteristics of the institutional environment and encompasses multiple dimensions, including activity, emotional feedback, freedom, privacy, safety, social structure, and support (Wright, 1985). These dimensions may be particularly relevant to inmates’ psychological well-being because supportive and predictable environments may provide resources for coping, whereas perceived environmental deprivation may be associated with greater psychological distress (Andrade et al., 2023; Megari, 2019).

Evidence from Nigeria indicates that suicidal ideation among inmates is associated with psychological distress, institutional conditions, social alienation, and other characteristics of incarceration (Amoke et al., 2020; Ndubisi et al., 2023; Olapegba et al., 2019; Olanrewaju and Akinyemi, 2021). International evidence similarly suggests that social support, perceived safety, isolation, and other correctional conditions are related to suicidal ideation and suicide risk among incarcerated populations (Favril et al., 2021; Fentahun et al., 2024). However, existing Nigerian studies have generally examined selected psychological, social, or institutional factors rather than systematically examining the seven dimensions of the Prison Environment Inventory (PEI) within a single analytical model. Consequently, it remains insufficiently understood how inmates’ perceptions of activity, emotional feedback, freedom, privacy, safety, social structure, and support are individually and jointly associated with suicidal ideation within Nigerian correctional settings.

The present study addresses this gap by examining the relationship between the seven dimensions of perceived correctional climate and suicidal ideation among inmates in Nigerian Correctional Centres. The study is informed by the Interpersonal Psychological Theory of Suicide, which proposes that suicidal ideation is associated with perceived burdensomeness and thwarted belongingness, while acquired capability relates to exposure to painful or provocative experiences (Joiner, 2005; Van Orden et al., 2010). From this perspective, dimensions such as emotional feedback and support may be conceptually relevant to belongingness, while privacy, safety, and meaningful activity may shape the broader environmental conditions within which inmates experience social connection, vulnerability, and psychological distress. These theoretical relationships provide a basis for examining correctional climate as a potential correlate of suicidal ideation, although the IPT constructs themselves are not directly measured in the present study.

Accordingly, this study examines whether the seven perceived correctional climate dimensions are significantly associated with suicidal ideation and identifies which dimensions demonstrate significant independent associations when considered simultaneously. By examining these dimensions collectively within the Nigerian correctional context, the study seeks to extend existing evidence and provide a more comprehensive understanding of environmental correlates of suicidal ideation among incarcerated individuals.

### Statement of the problem

The operational conditions within Nigerian correctional centres present inmates with significant psychological and environmental challenges, characterized by overcrowding, limited privacy, safety concerns, inadequate rehabilitation activities, and restricted psychosocial support (Amoke et al., 2020). This challenging environment correlates with a high prevalence of suicidal ideation among inmates, as documented in recent studies (Fentahun et al., 2024; Habtamu and Desalegn, 2022). However, despite the escalating evidence on suicide-related concerns in correctional facilities, there is a noteworthy lack of empirical research exploring how inmates’ perceptions of various elements of the correctional climate influence suicidal ideation specifically in Nigeria (Olapegba et al., 2019).

Current literature offers insufficient insights into whether certain dimensions of perceived correctional climate—such as activity, emotional feedback, freedom, privacy, safety, social structure, and support—have significant independent relationships with suicidal ideation when analyzed concurrently. Understanding these dimensions is crucial since the correctional climate is inherently multidimensional, and the impact of each dimension may vary. Consequently, this study aims to investigate both the joint and individual associations of these seven dimensions of perceived correctional climate with suicidal ideation among inmates in Nigerian Correctional Centres, addressing a critical gap in the existing body of knowledge.

### Objectives of the study

The study aims to explore both the joint and independent predictive role of perceived correctional climate dimensions on suicidal ideation among inmates in Nigerian Correctional Centres.

The specific objectives were:

1. To evaluate the predictive role of seven perceived dimensions of correctional climate (Activity, emotional feedback, freedom, privacy, safety, social structure, and support) on suicidal ideation among inmates in Nigerian Correctional Centres.

### Research hypotheses

To provide empirical structure for the investigation, the following alternate and directional hypotheses were formulated and tested at the 0.05 level of significance:

1. Higher levels of favourable perceptions of Activity will significantly predict lower suicidal ideation among inmates in Nigerian Correctional Centres.
2. Higher levels of favourable perceptions of Emotional Feedback will significantly predict lower suicidal ideation among inmates in Nigerian Correctional Centres.
3. Higher levels of favourable perceptions of Freedom will significantly predict lower suicidal ideation among inmates in Nigerian Correctional Centres.
4. Higher levels of favourable perceptions of Privacy will significantly predict lower suicidal ideation among inmates in Nigerian Correctional Centres.
5. Higher levels of favourable perceptions of Safety will significantly predict lower suicidal ideation among inmates in Nigerian Correctional Centres.
6. Higher levels of favourable perceptions of Social Structure will significantly predict lower suicidal ideation among inmates in Nigerian Correctional Centres.
7. Higher levels of favourable perceptions of Support will significantly predict lower suicidal ideation among inmates in Nigerian Correctional Centres.

### Conceptual clarifications

#### Suicidal ideation

Suicidal ideation encompasses thoughts and desires related to one’s own death or suicide, with varying degrees of intensity and severity, as described by Fentahun et al. (2024). This phenomenon can range from passive thoughts such as wishing not to exist, to active considerations of suicide. It is crucial to distinguish between suicidal ideation, suicidal intent (which indicates a commitment to act on these thoughts), and suicidal behavior (representing an actual suicide attempt) as outlined by Olapegba et al. (2019).

In correctional environments, the examination of suicidal ideation is exceptionally vital due to the various psychosocial stressors associated with incarceration. These stressors include limited personal autonomy, disruption of social connections, and heightened concerns regarding safety and mental wellbeing. Therefore, understanding suicidal ideation requires analyzing both the psychological traits of the individual and the broader environmental factors present within correctional facilities. In the current study, suicidal ideation serves as the focal point, evaluated through the Beck Scale for Suicide Ideation (BSS). The research aims to explore the relationship between the perceived correctional climate and suicidal ideation, intentionally setting aside considerations of suicidal intent or behavior.

#### Correctional climate

Correctional climate encompasses inmates’ perceptions of the psychological, social, and physical aspects of life within correctional institutions, as described by Wright (1985). It is a complex construct that captures how inmates evaluate their environment through various dimensions, including meaningful activities, emotional feedback, freedom, privacy, safety, social structure, and support. Each of these dimensions is crucial for understanding the institutional environment and the daily experiences of inmates.

Inmates’ perceptions can significantly differ based on their experiences of support, predictability, safety, and responsiveness to their psychosocial needs. A negative perception, characterized by restricted autonomy, inadequate support, limited privacy, or unsafe conditions, is often linked to adverse psychological outcomes (Megari, 2019). The current study operationalizes correctional climate using the seven dimensions from the Prison Environment Inventory (PEI), where higher scores reflect more positive perceptions of the environment. The focus of the research is on identifying environmental factors related to suicidal ideation among inmates, rather than establishing a direct causal relationship with such thoughts.

### Empirical review

The empirical review highlights the critical issue of suicidal ideation among correctional populations, emphasizing the interplay of individual vulnerabilities and institutional environmental factors. Studies by Fentahun et al. (2024) and Habtamu and Desalegn (2022) illustrate a high prevalence of suicidal thoughts among prisoners in Ethiopia, linking these ideations to inadequate social support and psychological distress. Such findings underscore the significance of both interpersonal relationships and the surrounding environment in understanding suicide risks within correctional settings. Further evidence from a Nigerian context reinforces the impact of the correctional climate on inmate mental health. Amoke et al. (2020) reveal a vital connection between perceived institutional conditions and coping mechanisms affecting suicidal ideation. Ndubisi et al. (2023) also note that environmental stressors, such as overcrowding and poor living conditions, contribute to psychological distress among awaiting-trial inmates, establishing a broader context for suicidal ideation. International studies corroborate these findings, emphasizing the relevance of safety, social support, and isolation as significant factors related to suicide outcomes (Favril et al., 2021; Barker et al., 2022).

Notably, research indicates that perceptions of correctional environments vary among inmates. Olapegba et al. (2019) identify disparities in suicidal ideation across different inmate categories, while Olanrewaju and Akinyemi (2021) connect an unsupportive environment and social alienation specifically with suicidal thoughts among female inmates. Such insights point to the need for a nuanced understanding of how individual characteristics and social experiences influence perceptions of correctional climate. Despite these insights, the Nigerian literature still has considerable gaps. Much of the existing research has primarily targeted specific institutional conditions or psychological traits without fully exploring the seven-dimensional structure of correctional climate, which includes activity, emotional feedback, freedom, privacy, safety, social structure, and support. The holistic associations between these dimensions and suicidal ideation remain underexplored, leading to an ambiguity regarding which aspects most significantly impact suicidal thoughts when analyzed together. The current study aims to fill this gap by investigating the seven dimensions of perceived correctional climate in relation to suicidal ideation among inmates in Nigerian Correctional Centres. Rather than positing a direct causal relationship, the study seeks to evaluate how inmates perceive these various environmental dimensions and their independent associations with suicidal ideation. This approach is designed to enhance the existing body of research in Nigeria by providing a comprehensive, multidimensional assessment of correctional climate, ultimately identifying specific environmental factors that significantly correlate with suicidal ideation.

### Theoretical framework

The study is grounded in Thomas Joiner’s Interpersonal Psychological Theory of Suicide (IPT), which posits that suicidal ideation results from the interplay of perceived burdensomeness, feeling like a burden to others and thwarted belongingness, which entails social disconnection, loneliness, and alienation. Additionally, IPT suggests that one’s capability for suicide develops through repeated exposure to distressing experiences, enhancing an individual’s tolerance for pain and fear of death. This theoretical framework aids in understanding suicidal thoughts among individuals in environments marked by social isolation and stress. The relevance of these constructs is particularly noted within correctional settings, where various dimensions of perceived correctional climate may correlate with IPT’s elements. Emotional feedback and institutional support are linked to inmates’ feelings of social connection and belonging, while the social structure and meaningful activities foster interpersonal interactions. Factors such as perceived privacy and safety contribute to inmates’ experiences of vulnerability and emotional distress. Moreover, the aspect of freedom is significant in understanding perceived autonomy in confinement. Nonetheless, the study does not directly measure the primary IPT mechanisms of perceived burdensomeness, thwarted belongingness, and acquired capability.

Operationalization of correctional climate in this research utilizes the seven dimensions outlined in the Prison Environment Inventory: activity, emotional feedback, freedom, privacy, safety, social structure, and support. These dimensions are analyzed for their associations with suicidal ideation; the latter being evaluated through the Beck Scale for Suicide Ideation. As such, IPT offers a conceptual basis for exploring how inmates’ perceptions of their social, psychological, and environmental circumstances relate to their thoughts of suicide, all while noting that the mechanisms proposed by the theory were not empirically tested in the current analysis.

## Method

### Study design and setting

This study employed a descriptive cross-sectional survey design to examine the relationship between perceived correctional climate and suicidal ideation among inmates. The study was conducted at Ibara Correctional Centre and New Abeokuta Correctional Centre (Oba), both located in Abeokuta, Ogun State, Nigeria. The New Abeokuta Correctional Centre is situated along Oba Road, Agbamaya, Abeokuta, and shares joint administrative programs and jail-delivery activities with Ibara Correctional Centre. The facilities were selected based on their accessibility for the study and the administrative arrangements that facilitated institutional access and data collection.

### Participants and sampling

Participants were 210 adult inmates selected using purposive sampling. Participants ranged in age from 21 to 60 years (*M* = 36.07, SD = 8.68). Of the participants, 118 (56.4%) were recruited from Ibara Correctional Centre and 92 (43.6%) from New Abeokuta Correctional Centre (Oba). The sample comprised 70.6% married, 11.8% single, and 17.5% divorced participants. Ethnically, 51.7% identified as Yoruba, 22.3% as Igbo, 2.4% as Hausa, and 23.7% as belonging to other ethnic groups. Most participants (80.6%) reported low socioeconomic status, while 19.4% reported moderate socioeconomic status.

Participants were eligible if they were adults incarcerated in the selected facilities, had been continuously incarcerated for at least 6 months, and had sufficient English literacy to understand and complete the study instruments. Participants who did not meet this eligibility requirements were not included. The sample size of 210 was considered adequate for the planned multiple linear regression involving seven predictor variables and exceeded the minimum sample size available for the study. Given the use of purposive sampling and the inclusion of participants from only two correctional facilities, the findings are interpreted within the context of the sampled population and should not be assumed to be representative of all inmates in Nigeria.

### Instruments

Perceived correctional climate was assessed using Wright’s (1985) 48-item Prison Environment Inventory (PEI), adapted to the study context. The adapted instrument comprised seven dimensions: Activity, Emotional Feedback, Freedom, Privacy, Safety, Social Structure, and Support. Items were rated on a 5-point Likert-type scale ranging from 1 (strongly disagree) to 5 (strongly agree). Negatively worded items were reverse-scored so that higher scores consistently indicated more favourable perceptions of the correctional environment. Scores for each dimension were obtained by summing the corresponding item responses, with higher scores reflecting more favourable perceptions of that dimension. Internal consistency was assessed separately for each dimension using Cronbach’s alpha, yielding coefficients of 0.78 for Activity, 0.81 for Emotional Feedback, 0.75 for Freedom, 0.84 for Privacy, 0.86 for Safety, 0.77 for Social Structure, and 0.83 for Support. The overall PEI demonstrated good internal consistency (*α* = 0.89).

Suicidal ideation was assessed using the Beck Scale for Suicide Ideation (BSS), developed by Beck et al. (1979). The BSS comprises 21 items, with the first 19 items scored to assess the intensity and severity of suicidal ideation; Items 20 and 21 provide supplementary information on previous suicide attempts. The 19 scored items are rated on a 3-point scale ranging from 0 to 2, with higher scores indicating greater severity of suicidal ideation. The BSS demonstrated good internal consistency in the present sample (Cronbach’s *α* = 0.84).

### Procedure and ethical considerations

Approval to conduct the study was obtained from the authorities of Ibara Correctional Centre and New Abeokuta Correctional Centre (Oba). Ethical approval was obtained from the Walter Sisulu University Research Ethics Committee following the required departmental and institutional review procedures. After institutional permission was secured, the researcher visited the correctional facilities and administered the questionnaires with assistance from the correctional welfare officer. Participants were informed about the purpose of the study, assured of confidentiality, and required to provide written informed consent before participation. Questionnaires were completed under supervised conditions to facilitate comprehension and appropriate completion of the instruments. Participation was voluntary, and confidentiality was maintained throughout the data-collection process.

### Statistical analysis

Data were analyzed using IBM SPSS Statistics version 30. Descriptive statistics were computed for the demographic and study variables. Multiple linear regression (MLR) was used to examine the extent to which the seven dimensions of perceived correctional climate independently predicted suicidal ideation. MLR was considered appropriate because the study sought to assess the unique contribution of multiple continuous predictor variables to a continuous outcome variable while controlling for the shared variance among the predictors.

The regression model was specified as:

SI=β0+β1ACT+β2EF+β3FR+β4PR+β5SA+β6SS+β7SU+USI

Where;

SI = Suicidal ideation.

ACT = Activity.

EF = Emotional Feedback.

FR = Freedom.

PR = Privacy.

SA = Safety.

SS = Social Structure.

SU = Support.

β₀ = Regression constant.

β₁ – β₇ Regression coefficients for the respective correctional climate dimensions.

U = error term.

The overall model was evaluated using *R*, *R*^2^, adjusted *R*^2^, and the F-statistic, while the independent contribution of each correctional climate dimension was assessed using the unstandardised regression coefficient (B), standardised regression coefficient (β), standard error (SE), *t*-value, *p*-value, and 95% confidence interval. Statistical significance was evaluated at the 0.05 level. The assumptions of linearity, normality of residuals, homoscedasticity, multicollinearity, and influential observations were assessed before interpreting the regression results. Given the cross-sectional design, the regression coefficients and model fit were interpreted as statistical associations and not as evidence of causal effects.

## Results

### Multiple linear regression of correctional climate and suicidal ideation

Prior to conducting and interpreting the regression model, the assumptions underlying multiple linear regression were assessed. Linearity was examined using scatterplots of the standardised predicted values against the standardised residuals, which indicated an approximately linear relationship between the predictors and suicidal ideation. The normality of the residuals was assessed using the normal probability (P–P) plot and histogram of standardised residuals, which showed that the residuals were approximately normally distributed. Homoscedasticity was assessed from the scatterplot of standardised residuals against standardised predicted values, which showed a reasonably random distribution of residuals around zero, with no clear evidence of systematic funneling.

Multicollinearity was assessed using tolerance and variance inflation factor (VIF) statistics. The tolerance values were estimated to range from 0.48 to 0.76, while the VIF values ranged from 1.32 to 2.08, indicating that multicollinearity was not a serious concern. Influential observations were assessed using Cook’s distance, with the maximum value estimated at 0.08, which was substantially below the commonly used criterion of 1.0. These diagnostic findings suggested that there were no serious violations of the assumptions of linearity, normality, homoscedasticity, multicollinearity, or influential observations. The data were therefore considered suitable for multiple linear regression analysis.

A multiple linear regression analysis was conducted to examine whether the seven dimensions of perceived correctional climate (activity, emotional feedback, freedom, privacy, safety, social structure, and support) were significantly associated with suicidal ideation among inmates. The overall regression model was statistically significant, *R* = 0.695, *R*^2^ = 0.483, adjusted *R^2^* = 0.465, *F* (7, 202) = 26.94, *p* < 0.001. The seven dimensions of perceived correctional climate jointly accounted for 48.3% of the variance in suicidal ideation.

As shown in Table 1, privacy had the strongest significant inverse association with suicidal ideation (*B* = −0.42, *β* = −0.34, *t* = −4.12, *p* < 0.001). Because higher scores on the Prison Environment Inventory (PEI) indicate more favourable perceptions of the correctional environment, this finding indicates that more favourable perceptions of privacy were associated with lower levels of suicidal ideation. Similarly, safety was significantly and inversely associated with suicidal ideation (*B* = −0.37, *β* = −0.29, *t* = −3.85, *p* < 0.001), indicating that more favourable perceptions of safety were associated with lower levels of suicidal ideation.

**Table 1:** Multiple linear regression analysis of correctional climate dimensions and suicidal ideation.

Predictor	*B*	SE	*β*	*t*	*p*	95% CI	
						Lower	upper
Constant	24.15	2.70		8.94	< 0.001	18.82	29.48
Activity	−0.18	0.09	−0.14	−1.98	0.049	−0.36	−0.01
Emotional feedback	−0.29	0.10	−0.22	−2.91	0.004	−0.49	−0.09
Freedom	−0.11	0.10	−0.09	−1.15	0.251	−0.30	0.08
Privacy	−0.42	0.10	−0.34	−4.12	< 0.001	−0.62	−0.22
Safety	−0.37	0.10	−0.29	−3.85	< 0.001	−0.56	−0.18
Social structure	−0.08	0.10	−0.06	−0.78	0.436	−0.28	0.12
Support	−0.24	0.10	−0.19	−2.46	0.015	−0.43	−0.05

*R* = 0.695; *R*^2^ = 0.483; Adjusted *R*^2^ = 0.465; *F* (7, 202) = 26.94, *p* < 0.001.

Emotional feedback was also significantly and inversely associated with suicidal ideation (*B* = −0.29, *β* = −0.22, *t* = −2.91, *p* = 0.004). This suggests that more favourable perceptions of emotional feedback were associated with lower levels of suicidal ideation. Support also showed a significant inverse association with suicidal ideation (*B* = −0.24, *β* = −0.19, *t* = −2.46, *p* = 0.015), indicating that more favourable perceptions of support were associated with lower suicidal ideation. Activity was likewise significantly and inversely associated with suicidal ideation (*B* = −0.18, *β* = −0.14, *t* = −1.98, *p* = 0.049). In contrast, freedom (*B* = −0.11, *β* = −0.09, *t* = −1.15, *p* = 0.251) and social structure (*B* = −0.08, *β* = −0.06, *t* = −0.78, *p* = 0.436) were not significantly associated with suicidal ideation in the multivariate model.

## Discussion

The present study examined the associations between seven dimensions of perceived correctional climate and suicidal ideation among inmates in Nigerian correctional centres. The regression model was statistically significant, with the seven dimensions jointly accounting for 48.3% of the observed variance in suicidal ideation. This finding suggests that inmates’ perceptions of their correctional environment are meaningfully associated with differences in suicidal ideation within the present sample. However, given the cross-sectional design, the findings should not be interpreted as evidence that correctional climate causes suicidal ideation.

Privacy emerged as the strongest independent predictor of suicidal ideation. Because higher PEI scores indicate more favourable perceptions of the correctional environment, the negative coefficient indicates that more favourable perceptions of privacy were associated with lower suicidal ideation. This finding is consistent with evidence that institutional conditions involving limited personal space, reduced autonomy, and inadequate privacy may be related to psychological distress among incarcerated populations (Andrade et al., 2023; Megari, 2019). In the Nigerian correctional context, restricted privacy may be particularly relevant where institutional resources and living conditions constrain inmates’ opportunities for personal space and psychological withdrawal.

Perceived safety was also significantly and inversely associated with suicidal ideation. Thus, inmates who reported more favourable perceptions of safety tended to report lower levels of suicidal ideation. This finding accords with literature linking exposure to threatening or unsafe correctional environments with psychological difficulties among incarcerated individuals (Fentahun et al., 2024). Rather than demonstrating that unsafe conditions cause suicidal ideation, the present finding suggests that perceived safety represents an important environmental correlate that warrants attention in correctional mental-health planning.

Emotional feedback, institutional support, and activity were likewise significantly and inversely associated with suicidal ideation. These findings suggest that more favourable perceptions of interpersonal responsiveness, institutional support, and meaningful activity corresponded with lower suicidal ideation. Such relationships are consistent with previous evidence emphasizing the relevance of social support, engagement, and institutional experiences to psychological well-being among incarcerated populations (Amoke et al., 2020; Olanrewaju and Akinyemi, 2021). In the Nigerian correctional context, opportunities for meaningful engagement and supportive interactions may provide inmates with social and psychological resources that are relevant to adjustment during incarceration.

Freedom and social structure did not demonstrate significant independent associations with suicidal ideation after the other correctional climate dimensions were considered simultaneously. This does not necessarily imply that these dimensions are irrelevant to inmate well-being. Rather, their independent contributions may be less pronounced when privacy, safety, emotional feedback, activity, and support are considered together. Differences between the present findings and previous studies may also reflect variations in institutional conditions, inmate characteristics, measurement approaches, and correctional systems across settings.

The findings can also be considered in relation to the Interpersonal Psychological Theory of Suicide (Joiner, 2005; Van Orden et al., 2010). From this perspective, correctional experiences involving limited social connection or inadequate support may be conceptually relevant to thwarted belongingness, while experiences of vulnerability or painful institutional conditions may be relevant to processes associated with acquired capability. Meaningful activity and supportive interpersonal interactions may likewise provide conditions that are theoretically relevant to social connectedness. However, because perceived burdensomeness, thwarted belongingness, and acquired capability were not directly measured in the present study, these relationships should be regarded as possible theoretical explanations rather than demonstrated psychological mechanisms.

The findings extend previous Nigerian evidence by considering seven dimensions of perceived correctional climate within a single multivariable model. The results indicate that privacy, safety, emotional feedback, support, and activity were the dimensions most strongly associated with suicidal ideation in the present sample. These findings highlight the importance of considering both psychosocial and environmental conditions when understanding suicidal ideation among incarcerated individuals in Nigeria.

## Limitations

Several limitations should be considered when interpreting the findings. These includes: the cross-sectional design which restricts causal inference; potential selection bias from purposive sampling; a small sample size of 210 inmates from two facilities limiting generalizability; reliance on self-report instruments susceptible to biases; exclusion of inmates with limited English proficiency; and the omission of individual psychological factors in the analysis.

Future research should aim for larger, diverse samples, longitudinal designs, better language access, and inclusion of psychological variables to enhance the evidence base. The study did not examine differences among inmate groups, such as gender, which could influence experiences in correctional environments and suicidal thoughts. It also did not assess broader correctional culture aspects. These elements may clarify variations in suicidal ideation but were not included in this study. Future research should examine inmate diversity and correctional culture alongside perceived climate to enhance understanding of suicidal ideation in Nigerian correctional settings.

## Conclusion

The study found that perceived correctional climate was significantly associated with suicidal ideation among inmates in the sampled Nigerian correctional centres. More favourable perceptions of privacy, safety, emotional feedback, institutional support, and activity were significantly associated with lower suicidal ideation, whereas freedom and social structure did not show significant independent associations in the multivariable model. The seven correctional climate dimensions jointly accounted for 48.3% of the observed variance in suicidal ideation in the present sample. These findings underscore the relevance of perceived environmental and interpersonal conditions to understanding suicidal ideation among incarcerated individuals. However, the cross-sectional design does not permit causal conclusions. Correctional mental-health strategies may therefore benefit from attention to privacy, safety, supportive interactions, and meaningful activities while recognizing that suicidal ideation is multifactorial and cannot be attributed to correctional climate alone.

### Implications for forensic psychology

These findings carry important implications for the theory and practice of forensic psychology within the West African legal and correctional systems. For clinical practice, the study highlights the necessity of incorporating objective environmental assessments into individual suicide risk evaluations, rather than relying solely on traditional psychiatric history. Administratively, it provides an evidence-based framework for designing institutional training programs that enhance mental health literacy among correctional officers, helping staff recognize early behavioral warning signs within high-risk units. Finally, for policy advocacy, these empirical indicators offer robust data to support legal and correctional system reforms that prioritize inmate welfare and mental health resources, helping align the Nigerian correctional network with international humanitarian standards.

### Recommendations

Based on the empirical findings of this study, the following recommendations are offered:

1. *Implement systematic mental health screening*: The Nigerian Correctional Service should integrate standardized mental health screening tools into admission protocols to identify vulnerable inmates and enable early clinical intervention.
2. *Execute structural and spatial reforms*: Administrative efforts should prioritize reducing cell overpopulation, optimizing spatial layout designs, and improving sanitation standards to preserve basic levels of inmate privacy and safety.
3. *Expand rehabilitation and coping programs*: Facilities should increase access to structured educational, vocational, and recreational activities, alongside evidence-based counseling interventions like Cognitive Behavioral Therapy (CBT), to build adaptive distress tolerance and coping skills among inmates.

## Data Availability

The original contributions presented in the study are included in the article/supplementary material, further inquiries can be directed to the corresponding author/s.
